# Sulfur Vulcanization and Material Properties of Polyhydroxyalkanoates with Unsaturated Side Chain

**DOI:** 10.3390/polym17182561

**Published:** 2025-09-22

**Authors:** Phimthong Khamjapo, Lucas Vinicius Santini Ceneviva, Yusuke Nakata, Yuki Miyahara, Takeharu Tsuge

**Affiliations:** Department of Materials Science and Engineering, Institute of Science Tokyo, 4259 Nagatsuta, Midori-ku, Yokohama 226-8501, Japan

**Keywords:** sulfur vulcanization, crosslinking, polyhydroxyalkanoates, unsaturated side chain, vinyl copolymer

## Abstract

This study aimed to evaluate the physical properties and biodegradability of sulfur-vulcanized polyhydroxyalkanoates (PHAs) with unsaturated side chains. As a vulcanizable PHA, poly(3-hydroxybutyrate-*co*-3-hydroxy-5-hexenoate) [P(3HB-*co*-3H5HE)] was biosynthesized with a 3H5HE fraction of 3–47 mol% using recombinant *Escherichia coli* and subsequently vulcanized with varying sulfur contents (2–20 per hundred resin, phr) in the presence of zinc oxide, stearic acid, and 2-mercaptobenzothiazole as curing agents. The vulcanized PHA copolymers were insoluble in chloroform, indicating the formation of a cross-linked network. Raman spectroscopy revealed the functional loss of the double bonds in the polymers. After the vulcanization with 5 phr sulfur, the tensile strength and elongation at break of P(3HB-*co*-47 mol% 3H5HE) increased from 0.6 MPa to 6.3 MPa and from 430% to 813%, respectively. This sample exhibited low tensile set (8%) after 200% elongation, indicating rubber-like properties. Although biodegradability decreased with increasing crosslink density, vulcanized P(3HB-*co*-3H5HE) exhibited a greater degradation potential than vulcanized rubber but was lower than that of non-vulcanized P(3HB-*co*-3H5HE). These findings demonstrate that sulfur vulcanization can enhance the resilience of unsaturated PHAs, making them suitable for elastomeric and environmental applications.

## 1. Introduction

Polyhydroxyalkanoates (PHAs) are a family of polyesters biosynthesized by various bacterial species as intracellular carbon and energy storage materials. Because of their biodegradability and renewability, PHAs have attracted significant interest as sustainable alternatives to petroleum-based plastics [1,2,3]. Among these, poly(3-hydroxybutyrate) [P(3HB)] is the most typical naturally occurring PHA. However, owing to its high crystallinity, P(3HB) is inherently brittle and exhibits poor flexibility and impact resistance, which greatly restricts its practical application, especially in areas requiring mechanical toughness and elasticity [1,2,3]. The tensile strength and elongation at break of P(3HB) are recorded as 40 MPa and 5%, respectively [2].

To overcome these limitations of P(3HB), various 3HB-based copolymers have been developed [2,3,4,5,6,7,8]. For example, P(3HB-*co*-3-hydroxyvalerate) [P(3HB-*co*-3HV)] and P(3HB-*co*-3-hydroxyhexanoate) [P(3HB-*co*-3HHx)] have demonstrated enhanced elongation at break and reduced crystallinity by preventing the formation of P(3HB) crystals through the introduction of comonomer units with bulky side chains [4,5,6,7]. The values of elongation at break for P(3HB-*co*-20 mol% 3HV) and P(3HB-*co*-10 mol% 3HHx) were recorded at 50% and 400%, respectively [2,7]. Copolymerization alters the mechanical performance of the PHA materials, thereby rendering them more practical. Various hydroxyalkanoate monomers are known to have been incorporated into PHA; however, only PHAs with hydrophobic side chains can be polymerized because of substrate recognition by PHA-polymerizing enzymes (PHA synthases) [9].

Some naturally occurring PHAs contain unsaturated bonds in their side chains [10,11]. *Pseudomonas* spp. are known to synthesize medium-chain-length PHAs containing unsaturated monomers such as 3-hydroxy-5-dodecenoate and 3-hydroxy-5-tetradecenoate [10]. In contrast, *Rhodospirillum rubrum* and *Burkholderia* sp. can polymerize short-chain PHAs containing unsaturated side chains [12,13]. By chemically modifying the unsaturated side chains of PHAs with various functional groups using techniques such as thiol-ene click chemistry, epoxidation, hydrosilylation, and free radical grafting, new functionalities can be introduced into these polymers [14,15,16,17,18,19,20]. In our previous study, we showed that polyethylene glycol grafted onto the unsaturated side chain of PHA conferred antifouling ability to its surface, thereby altering its biodegradability [18]. Thus, unsaturated side-chain monomers expand the applications of PHA by providing sites for chemical modification.

Polyisoprene, a natural unsaturated hydrocarbon, exhibits exceptional mechanical strength and elastic properties when vulcanized with sulfur [21,22,23,24,25]. Sulfur vulcanization is a well-established technique in elastomer processing that involves the formation of covalent cross-links between polymer chains. However, the biodegradability of vulcanized polyisoprene is drastically reduced compared to that of non-vulcanized polyisoprene [26,27,28]. For medium-chain-length (mcl) PHA containing unsaturated side chains, sulfur vulcanization has also been conducted to enhance the elastic response of polymers, imparting rubber-like elasticity to the PHA [24]. However, no study has been conducted on the sulfur vulcanization of 3HB-based vinyl copolymers. It is of interest to understand the material and biodegradability properties of 3HB-based vinyl copolymers when vulcanized with sulfur because 3HB-based polymers show different physical properties from mcl-PHA [2] and excellent biodegradability in natural environments [29,30,31].

In this study, poly(3-hydroxybutyrate-*co*-3-hydroxy-5-hexenoate) [P(3HB-*co*-3H5HE)] was synthesized as a 3HB-based vinyl copolymer by incorporating alkene functional groups along its side chains. P(3HB-*co*-3H5HE) exhibited greater flexibility than P(3HB) but displayed a lower tensile strength, resulting in lower toughness. The transformation of PHA results in a more ductile and resilient polymer that is suitable for a broad range of practical applications. Additionally, the effect of sulfur vulcanization on the biodegradability of PHA has not yet been investigated. Therefore, in this study, sulfur vulcanization of P(3HB-*co*-3H5HE) was conducted, which altered its mechanical properties. Then, the physical and biodegradation properties of the resulting vulcanized polymers were evaluated.

## 2. Materials and Methods

### 2.1. Materials

Sulfur, ZnO, and stearic acid were purchased from Kanto Chemical Co., Inc. (Tokyo, Japan). 2-Mercaptobenzothiazole (MBT) was purchased from Tokyo Chemical Industry Co., Ltd. (Tokyo, Japan). P(3HB-*co*-5 mol% 3HHx) was kindly provided by the KANEKA Corporation (Osaka, Japan).

### 2.2. Bacterial Strain and Plasmid

For P(3HB-*co*-3H5HE) biosynthesis, *Escherichia coli* LS5218 harboring the plasmid pBBR1*phaP*(D4N)*JC*_Ac_(NSDG)*AB*_Re_ [18,32,33] was used as the production host. *E. coli* LS5218 is a *fadR601 atoC2*(Con) mutant. The product of the *fadR* gene (FadR) is a transcription factor that negatively regulates fatty acid β-oxidation [34]. The *atoC2*(Con) is a constitutively active mutation for the response regulator gene (*atoC*). These mutations allow for the constitutive expression of enzymes involved in the utilization of fatty acids.

### 2.3. PHA Biosynthesis and Extraction

The recombinant *E. coli* LS5218 was pre-cultured in test tubes containing 10 mL of LB medium (10 g/L tryptone, 5 g/L bacto-yeast extract, and 10 g/L NaCl) at 30 °C and 130 rpm for 15 h. The pre-culture (1 vol%) was then inoculated to a 1000 mL M9-modified medium (17.1 g/L Na_2_HPO_4_·12H_2_O, 3 g/L KH_2_PO_4_, 0.5 g/L NaCl, 2.5 g/L yeast extract, 2 mL/L 1 M MgSO_4_·7H_2_O, 100 µL/L 1 M CaCl_2_) supplemented with 1.5 g/L sodium 5-hexenoate, 1 mM isopropyl-*β*-_D_-thiogalactopyranoside (IPTG), and glucose at the following concentrations (Table 1): For P(3HB-*co*-3 mol% 3H5HE) biosynthesis, 10 g/L glucose was added at the beginning of the cultivation. For P(3HB-*co*-19 mol% 3H5HE) biosynthesis, 11.25 g/L of glucose was added in three portions (3.75 g/L at 0, 24, and 48 h). For P(3HB-*co*-47 mol% 3H5HE) biosynthesis, 7.5 g/L of glucose was added in three portions (2.5 g/L at 0, 24, and 48 h). All cultures were incubated in 2 L shake flasks at 30 °C and 103 rpm for 72 h.

Following cultivation, the culture broth was centrifuged at 25 °C, 4220× *g* for 10 min to separate the bacterial cells. The culture supernatant was discarded, and the resulting bacterial pellet was resuspended in distilled water. The centrifugation was repeated. The washing step was repeated twice to remove the residual medium components and bacterial metabolites. The bacterial cells were then freeze-dried under vacuum for 72 h.

Dried bacterial cells and chloroform were added to a glass bottle and stirred at 50 °C for 24 to 48 h using a hot stirrer to extract the polymer. After extraction, the polymer solution was filtered under reduced pressure through a filter paper (No. 1; Advantec, Tokyo, Japan) that had been overlaid with a layer of diatomaceous earth (Celite 545; Kanto Chemical Co., Inc., Tokyo, Japan) to remove the cells. The filtrate was then concentrated by removing the chloroform using a rotary evaporator, resulting in a concentrated PHA solution. The concentrated PHA solution was slowly dropped into stirred methanol to precipitate the polymer. The precipitated PHA was collected by filtration using a filter paper (No. 5B, Advantec), followed by drying in a vacuum dryer to obtain purified PHA.

### 2.4. Nuclear Magnetic Resonance Spectroscopy

The chemical structure of the biosynthesized polymer was analyzed using ^1^H nuclear magnetic resonance (NMR) spectroscopy. A polymer sample (10–30 mg) was placed in a 5 mL vial and dissolved in approximately 1 mL of deuterochloroform (CDCl_3_) containing tetramethylsilane. The polymer solution was transferred to an NMR tube to prepare the NMR samples. The NMR instrument used was an AVANCE III 400 (Bruker Biospin GmbH, Ettlingen, Germany).

### 2.5. Compound Preparation and Testing

The formulations used for sulfur vulcanization are listed in Table 2. The vulcanization process for making the compound was conducted through a sequential mixing of additives into the copolymer matrix in a round-bottom flask with a stirrer bar at a temperature of 50 °C and a speed of 20 rpm using a stirring mantle. Initially, the PHAs were masticated for 1 min to soften the material and prepare it for compounding. ZnO was then added and mixed for 1 min, followed by the incorporation of stearic acid and further mixing for 1 min. Subsequently, MBT was added, and the mixture was mixed for 1 min. Finally, sulfur was added and compounded over a total mixing time of 10 min. The compound was recovered from the flask, and the mixture was pre-formed into sheets using a hydraulic hot press at 75 °C for 15 s, followed by manual rolling. This step was repeated ten times to ensure uniform dispersion of all curatives. The processed sheets were then left to stabilize for two days. Finally, vulcanized films were fabricated by compression molding at 155 °C for each cure time using a hydraulic hot press. After the fabrication process, the vulcanized films were left at room temperature for at least four days before characterizing their properties.

### 2.6. Molecular Weight Analysis

The molecular weight was determined by gel permeation chromatography (GPC) using a Shimadzu Nexera 40 GPC system (Kyoto, Japan) and Shodex RI-504 refractive index detector (Showa Denko, Tokyo, Japan). The separation was performed with one Shodex GPC KF-G 4A column and two GPC KF-406LHQ columns in a column oven at 40 °C. The eluent was HPLC-grade chloroform flowing at a rate of 0.3 mL/min. Calibration curves were generated using polystyrene standards with low polydispersity.

### 2.7. Thermal Property Analysis

The thermal properties of the sample were characterized using a differential scanning calorimetry (DSC) device, DSC 8500 (Perkin-Elmer, Waltham, MA, USA), under a helium atmosphere. Each sample was held at −50 °C for 1 min before the heating cycle. The first heating was conducted from −50 °C to 200 °C at a rate of 20 °C/min, followed by a 1 min hold. The sample was then rapidly cooled using liquid nitrogen from 200 °C to −50 °C at a cooling rate of 500 °C/min and held for 1 min. Subsequently, a second heating scan was performed from −50 °C to 200 °C at a heating rate of 20 °C/min, with an additional 1 min hold. Finally, a cooling scan was performed on the sample using liquid nitrogen. The sample was cooled from 200 °C to −50 °C at a rate of 20 °C/min, followed by a 1 min hold. During the first heating scan, the melting temperature (*T_m_*) and the enthalpy of fusion (Δ*H_m_*) were recorded. A second heating scan was performed to determine the glass transition temperature (*T_g_*) and cold crystallization temperature (*T_cc_*). The crystallization temperature (*T_c_*) was determined from the cooling scan.

Thermogravimetric analysis (TGA) was performed using a Shimadzu DTG-60 instrument under nitrogen atmosphere. The weight loss of the sample was recorded over a temperature range of 20 to 500 °C under controlled heating conditions.

### 2.8. Mechanical Property Analysis

The mechanical properties of the sample were evaluated using a Shimadzu EZ-S 500N universal testing machine at a constant strain rate of 5 mm/min. The measured parameters included tensile strength, elongation at break, Young’s modulus, toughness, and tensile set. The tests were conducted on dumbbell-shaped specimens with gauge lengths of 10 mm, widths of 2 mm, and film thicknesses of approximately 0.05 mm. The dumbbell-shaped specimens were cut using a super dumbbell cutter (SDMP-1000, Dumbbell Co., Ltd., Saitama, Japan).

### 2.9. Raman Spectroscopy Analysis

Raman spectroscopy was performed using a PR-1w palmtop Raman spectrometer (Jasco, Tokyo, Japan). Raman spectral data were collected over the range of 200 to 3000 cm^−1^ using a 785 nm excitation laser operating at 50 mW, with an exposure time of 1 s and 64 accumulations. The measurements were performed to enable a comparative analysis of the chemical structure of the polymer before and after vulcanization, with particular emphasis on the changes in the concentration of carbon-carbon double bonds.

### 2.10. Equilibrium Swelling Test

A swelling test was conducted by measuring the weights of the samples before immersion, after swelling in chloroform, and after drying. The samples were immersed in 10 mL of chloroform in sealed vials and kept at room temperature in the dark for 7 days. After the swelling period, the samples were removed and dried in a fume hood for 1 day to allow the complete evaporation of the residual solvent. The swelling ratio and gel fraction were calculated using the following equations [35]:

Swelling ratio (%) = 100 × (*M_s_ − M_i_*)/*M_i_*

where *M_i_* is the initial dry mass of the film and *M_s_* is the mass of a fully swollen film.

Gel fraction (%) = 100 × *W_f_*/*W_i_*

where *W_i_* is the initial mass of the film and *W_f_* is the final dried mass of the swollen films after immersion in the solvent.

### 2.11. Elemental Composition Analysis

Elemental analysis was performed to determine the compositions of the samples. The carbon, hydrogen, nitrogen, and ash contents were measured using a JM10 microcorder (J-Science Lab, Kyoto, Japan), while the oxygen content was quantified using an Elementer Vario Micro Cube. The sulfur content was measured separately using an HSU-20 (Yanaco, Kyoto, Japan). For the vulcanized samples, the oxygen content was calculated indirectly because of the presence of a large amount of ash.

### 2.12. Biodegradation Test

Biodegradability was assessed based on biochemical oxygen demand (BOD) using BOD instrumentation (OxiTop-IDS measuring head with a 250 mL BOD reactor, WTW GmbH, Weilheim, Germany). Pond water collected on 1st May 2025 (the highest and lowest temperatures of the day were 22.7 °C and 13.7 °C, respectively), from the Suzukakedai Campus (Nagatsuta, Yokohama City, 35°30′53.4″ N 139°29′06.3″ E), the Institute of Science Tokyo, was used. Test pond water was filtered through cotton and aerated using air compressor to consume the remaining carbon sources by existing microbes in the water. The pond water used in the BOD test contained approximately 1 g/L of pond sediments as inoculum. Ammonium chloride (0.5 g/L), KH_2_PO_4_ (0.1 g/L), and allylthiourea (20 drops of 5 g/L solution in 1 L of medium) were added to the pond water to inhibit nitrification. Pond water (150 mL) was placed in each of the 250 mL BOD measuring devices. Approximately 15 mg of a film sample (cut into 5 × 5 mm pieces) was used in the BOD test. The experiments were performed in quadruplicate. The biodegradability was defined as follows:

BOD biodegradability (%) = 100 × (BOD_s_ − BOD_b_)/ThOD

where BOD_s_, BOD_b_, and ThOD are the experimentally observed oxygen demands of the sample, blank, and theoretically calculated values, respectively. The BOD biodegradation tests were conducted at 20 °C [36].

## 3. Results

### 3.1. Biosynthesis and Structural Analysis of P(3HB-co-3H5HE)

**Figure 1:**
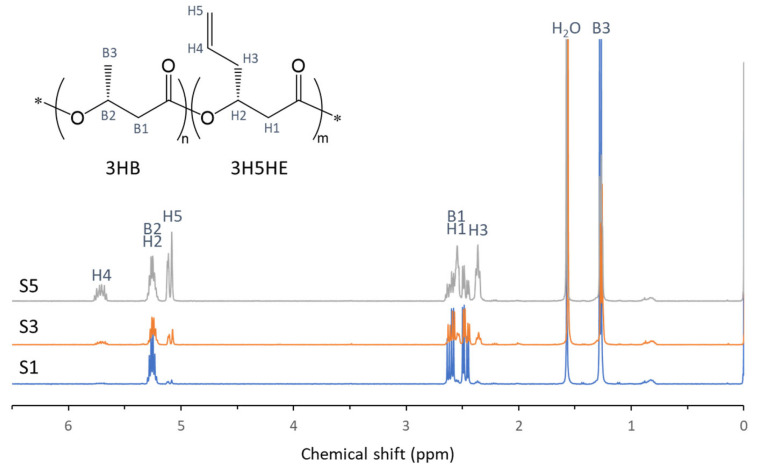
The 400 MHz ^1^H NMR spectra of P(3HB-*co*-3H5HE). The 3H5HE fractions of samples S1, S3, and S5 are 3 mol%, 19 mol%, and 47 mol%, respectively.

P(3HB-*co*-3H5HE) was biosynthesized as a 3HB-based vinyl copolymer using sodium 5-hexenoate as the 3H5HE precursor. Table 1 summarizes the copolymer composition and molecular weight of the biosynthesized P(3HB-*co*-3H5HE). The 3H5HE fraction in PHA was varied by controlling the glucose supply during culture. The resulting 3H5HE fraction in the copolymer was 3–47 mol%. The ^1^H NMR spectrum and signal assignments of P(3HB-*co*-47 mol% 3H5HE) are shown in Figure 1. 

All the biosynthesized polymers contained small amounts of 3HHx units (1 mol% or less), which will be ignored hereafter. The molecular weights of the biosynthesized P(3HB-*co*-3H5HE) ranged from 3.98 × 10^4^ to 15.4 × 10^4^ with a relatively large polydispersity index (PDI, 2.33–4.53).

### 3.2. Film Appearance and Solvent Solubility of Vulcanized P(3HB-co-3H5HE)

**Figure 2:**
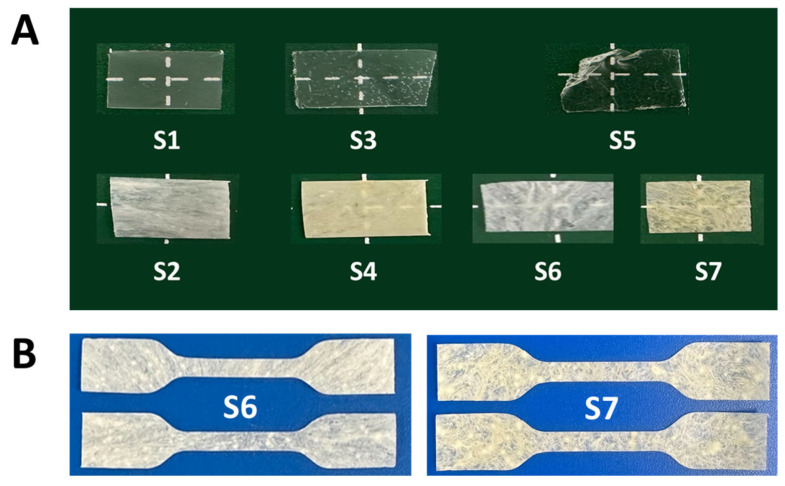
(**A**) Appearance of P(3HB-*co*-3H5HE) and vulcanized P(3HB-*co*-3H5HE) films (approximately 10 mm × 5 mm × 0.05 mm). (**B**) Dumbbell-shaped film (gauge length: 10 mm, width: 2 mm, film thickness: approximately 0.05 mm) for mechanical property measurements (samples S6 and S7).

The PHA copolymers were vulcanized under the conditions listed in Table 2. The appearances of both the non-vulcanized and vulcanized samples with varying molar ratios of 3H5HE units are shown in Figure 2A. Differences between the transparency, surface uniformity, and film integrity of the samples were observed, highlighting the influence of the copolymer composition and additive content on the polymer morphology. The non-vulcanized samples exhibited high transparency and homogeneous surfaces, indicating that the films were well-formed. However, the film containing 19 mol% 3H5HE appeared more brittle, whereas the 47 mol% 3H5HE sample exhibited a sticky texture, suggesting poor film stability at high unsaturation.

For the vulcanized samples, a decrease in transparency was observed. Vulcanization introduces crosslinking, leading to the formation of a three-dimensional network interconnected by sulfur bridges. The molecular arrangement and bonding within this network scatter and absorb incident light, thereby restricting its transmission and resulting in an opaque appearance. With increasing sulfur content, the films became progressively more opaque and rigid, indicating an increase in the crosslinking density and network formation within the polymer matrix.

Non-vulcanized P(3HB-*co*-3H5HE) is soluble in organic solvents, particularly chloroform. However, upon introduction of cross-linking through vulcanization, the polymer lost its solubility (Table 3). These results indicate the formation of a crosslinked network between the polymer chains.

### 3.3. Mechanical Properties

**Figure 3:**
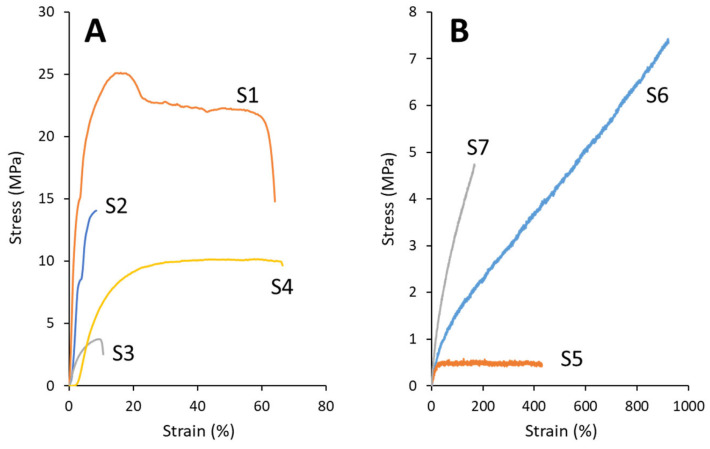
Representative stress–strain curves of P(3HB-*co*-3H5HE) and vulcanized P(3HB-*co*-3H5HE). (**A**) Samples S1 to S4. (**B**) Samples S5 to S7.

Dumbbell-shaped samples were prepared for mechanical property measurements, as shown in Figure 2B. The mechanical properties, including the tensile strength, elongation at break, Young’s modulus, and toughness, of the vulcanized and non-vulcanized films are summarized in Table 3. The stress–strain curves of these samples are presented in Figure 3.

**Table 3:** Mechanical properties and solvent solubility of the samples.

Sample	Solubility in Chloroform	Mechanical Properties			
		Tensile Strength (MPa)	Elongation at Break (%)	Young’s Modulus (MPa)	Toughness (MJ/m^3^)
S1	Dissolved	25	64	475	14
S2	Not Dissolved	14	9	238	0.78
S3	Dissolved	3.1 ± 1.0	9.4 ± 1.0	43 ± 31	0.15 ± 0.13
S4	Not Dissolved	9.9 ± 1.0	73 ± 14	49 ± 21	5.8 ± 1.1
S5	Dissolved	0.6	430	1.9	2.0
S6	Not Dissolved	6.3 ± 1.0	813 ± 94	2.6 ± 0.2	25 ± 10
S7	Not Dissolved	4.1 ± 0.6	135 ± 29	6.0 ± 0.1	3.4 ± 1.2

Repeated measurements were performed three times (*n* = 3), and the other measurements were performed once.

For sample S1, the low 3H5HE content polymer exhibited the highest tensile strength (25 MPa) and Young’s modulus (475 MPa), indicating that it was the most rigid and stiff material. After vulcanization, the tensile strength of the low 3H5HE-content polymer (S2) became relatively high (14 MPa), but it was lower than that of the non-vulcanized sample (S1). However, the elongation at break and Young’s modulus of S2 were 9% and 238 MPa, respectively, indicating its brittle behavior and stiffness.

For sample S3 (non-vulcanized 19 mol% 3H5HE copolymer), the tensile strength and elongation at break were 3.1 MPa and 9.4%, respectively, indicating brittle and stiff behaviors. However, after vulcanization (S4), the tensile strength and elongation at break were increased to 9.9 MPa and 73%, respectively. This shows that vulcanization can alter the mechanical properties.

Sample S5 (non-vulcanized 47 mol% 3H5HE copolymer) was the weakest, with a low tensile strength (0.6 MPa) and large elongation at break (430%). These values indicate that S5 is a soft and ductile material. Sample S6 exhibited the highest elongation at break (813%), indicating high flexibility and ductility, typical of elastomeric or rubber-like materials. Additionally, S6 exhibited a tensile set of 8 ± 3% (*n* = 3) after 200% elongation, which indicates excellent elasticity [24]. However, the tensile strength and elongation at break of S7 decreased to 4.1 MPa and 135%, respectively, when the sulfur content was increased for vulcanization.

### 3.4. Thermal Properties

The thermal properties of samples S1, S3, S5, S6, and S7 are summarized in Table 4. All measured samples exhibited a single sharp melting peak, indicating the formation of a relatively uniform crystalline thickness. As the molar fraction of 3H5HE increased, the *T_m_* value gradually shifted to lower temperatures, suggesting that the unsaturated 3H5HE units disrupted the regularity of the polymer chain and reduced crystallinity. A comparison of the non-vulcanized and vulcanized samples showed that the melting peak of S6 (containing 5 phr sulfur) appeared at a lower temperature than that of S5 (non-vulcanized), whereas S7 (containing 20 phr sulfur) exhibited a slightly higher melting temperature. These shifts imply that the extent of crosslinking influences the thermal behavior. Furthermore, the Δ*H_m_* of S5, which had a high 3H5HE content, was significantly lower than that of S1, confirming a reduction in crystallinity with increased unsaturation. Vulcanization with 5 phr sulfur (S6) led to a slight increase in crystallinity compared with that of S5. In contrast, excessive crosslinking in S7 (20 phr sulfur) appeared to suppress crystallinity, as reflected by its lower Δ*H_m_*.

The second DSC heating revealed that the *T_g_* value decreased with increasing 3H5HE fraction, indicating enhanced flexibility due to the disruption of chain packing in the amorphous region. Interestingly, after vulcanization, the *T_g_* values increased slightly, which may be attributed to the restricted chain mobility caused by the formation of cross-linked networks. The *T_cc_* value also increased with increasing 3H5HE fraction. Sample S1 demonstrated a lower *T_cc_* than sample S5, indicating a faster crystallization ability compared to S5. However, S6 and S7 exhibited better crystallization behaviors than S5.

In the cooling scans, Sample S1 exhibited a high *T_c_* of 92 °C, indicating a strong crystallization ability. In contrast, *T_c_* was not detected for samples S5, S6, and S7, suggesting that when the 3H5HE fraction reached 47 mol%, the copolymer exhibited extremely slow crystallization kinetics.

### 3.5. Thermogravimetric Analysis

**Figure 4:**
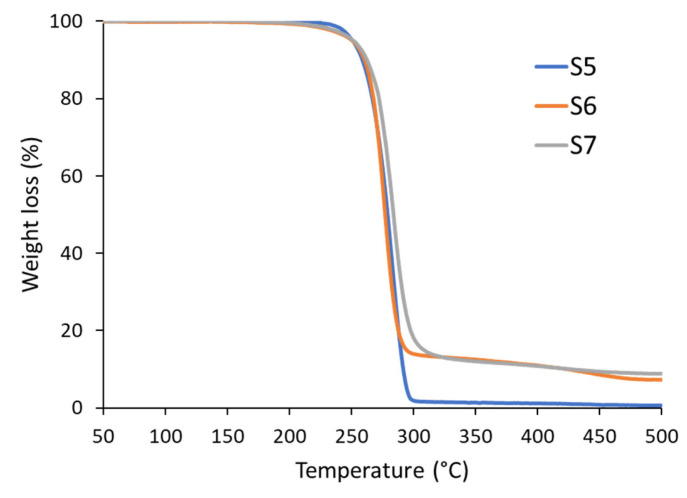
Thermogravimetry analysis (TGA) curves of samples S5, S6, and S7.

The thermal stabilities of S5, S6, and S7 were analyzed using TGA, as shown in Figure 4. The maximum degradation temperatures, residual mass percentages, and weight losses at 5% were determined as listed in Table 4. The TGA curves show weight loss in two stages for all three samples (S5, S6, and S7). The primary step of degradation of the polymer occurred above 170 °C, which was evident over the sample’s melting point. Sample S7 exhibited the highest *T_d_max_* of 331 °C, indicating greater thermal stability compared to that of S5 (319 °C) and S6 (312 °C). This suggests that the crosslinking in S7 likely enhances its resistance to thermal breakdown. Furthermore, the residual mass of S5 was negligible (1%), whereas S6 and S7 showed higher residual masses of 7% and 9%, respectively. This could indicate the presence of inorganic additives, sulfur residues from vulcanization, or other impurities that remained undecomposed after heating.

### 3.6. Raman Spectroscopy

**Figure 5:**
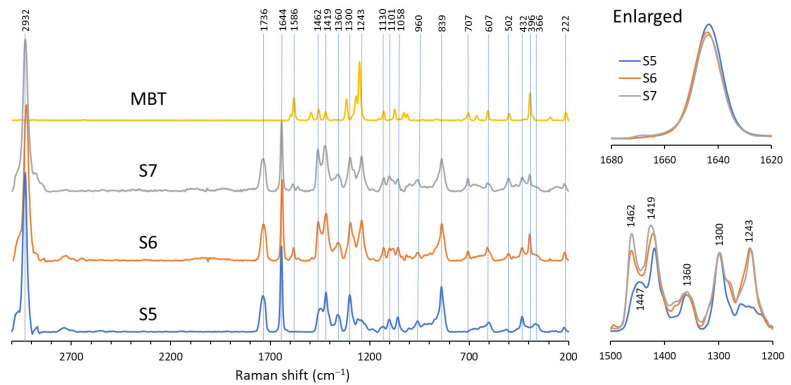
Raman spectra of samples S5, S6, S7, and MBT.

To analyze the vulcanization progress of S5, S6, and S7, the vinyl groups in the polymers were monitored using Raman spectroscopy, as shown in Figure 5. All spectra were normalized to the band at 1736 cm^−1^, corresponding to the C=O stretching vibration in the main chain of the P(3HB-*co*-3H5HE). The signal at 2932 cm^−1^ is attributed to C-H bond stretching, whereas the band at 1644 cm^−1^ is assigned to C=C stretching originating from unsaturated 3H5HE units. After vulcanization, a new signal emerged at 1586 cm^−1^, corresponding to aromatic C=C from the vulcanization accelerator MBT. Additional characteristic signals observed at 1419 cm^−1^, 1447 cm^−1^, and 1462 cm^−1^ are attributed to CH_3_ symmetric deformation, CH_2_ deformations, and CH_3_ asymmetric deformation, respectively. The signal at 1300 cm^−1^ corresponds to CH deformation (amorphous), whereas the signal at 1360 cm^−1^ corresponds to CH deformation (crystalline) and CH_3_ symmetric deformation. A signal at 1243 cm^−1^ was enhanced after sulfur vulcanization, probably due to the presence of new vibrational modes related to crosslinking. The signal at 960 cm^−1^ is assigned to C-C stretching. Furthermore, the signals at 396 cm^−1^, 707 cm^−1^, and 1130 cm^−1^ were observed in vulcanized samples, corresponding to CCO deformation (crystalline), C=O deformation (out-of-plane), and CH_3_ asymmetric rocking, respectively. The signals at 222 cm^−1^, 432 cm^−1^, 502 cm^−1^, 607 cm^−1^, and 839 cm^−1^ correspond to CH_3_ torsion, CCO deformation, C-CH_3_ and CCO deformation, C-CH_3_ and CCO deformation, and C-COO stretching, respectively [37,38].

A decrease in the peak area of the C=C band at 1644 cm^−1^ was observed after vulcanization, as listed in Table 5. These peak areas of samples S6 and S7 were calculated to be 87.8% and 88.4%, respectively, compared to that of S5. This indicates that most of the unsaturated bonds in 3H5HE units remain even after vulcanization.

### 3.7. Equilibrium Swelling Behavior

Non-vulcanized P(3HB-*co*-3H5HE) is soluble in organic solvents, particularly chloroform. However, upon the introduction of cross-linking via vulcanization, the polymer lost its solubility. The swelling ratio and gel fraction were determined to evaluate the degree of crosslinking (Table 5). For sample S6, a high swelling ratio of 655% was observed, indicating a crosslinked network capable of absorbing a substantial amount of solvent. A gel fraction of 93% confirmed that the majority of the polymers successfully formed an insoluble cross-linked network. In contrast, increasing the sulfur content to 20 phr (S7) resulted in a reduction in the swelling ratio to 338%. Coupled with a comparably high gel fraction of 94%, this strongly suggests that a higher sulfur loading and longer curing time led to a substantial increase in the crosslinking density. A higher crosslinking density restricts the ability of the polymer chain to expand and absorb the solvent, thereby reducing the swelling ratio.

### 3.8. Elemental Composition of Samples

The elemental compositions of S5, S6, and S7 are listed in Table 5. Sample S5, which represents a non-vulcanized polymer, was devoid of sulfur atoms. A significant increase (2.8 wt%) in the sulfur content of S6 was observed. The ash content also increased to 4 wt%, suggesting the presence of inorganic residues such as zinc. Further increasing the sulfur loading to 20 phr (S7) resulted in an increase in the sulfur content to 7 wt%. Additionally, a small amount of nitrogen was detected in the vulcanized P(3HB-*co*-3H5HE), which was attributed to the presence of MBT as an accelerator in the vulcanization reaction. Based on the elemental analysis, the composition formulas of samples S5, S6, and S7 were calculated to be C_4.98_H_6.87_O_2.04_, C_4.72_H_6.7_N_0.03_O_1.84_S_0.09_, and C_4.53_H_6.48_N_0.03_O_1.71_S_0.22_, respectively.

### 3.9. Biodegradability Test of Vulcanized PHA

**Figure 6:**
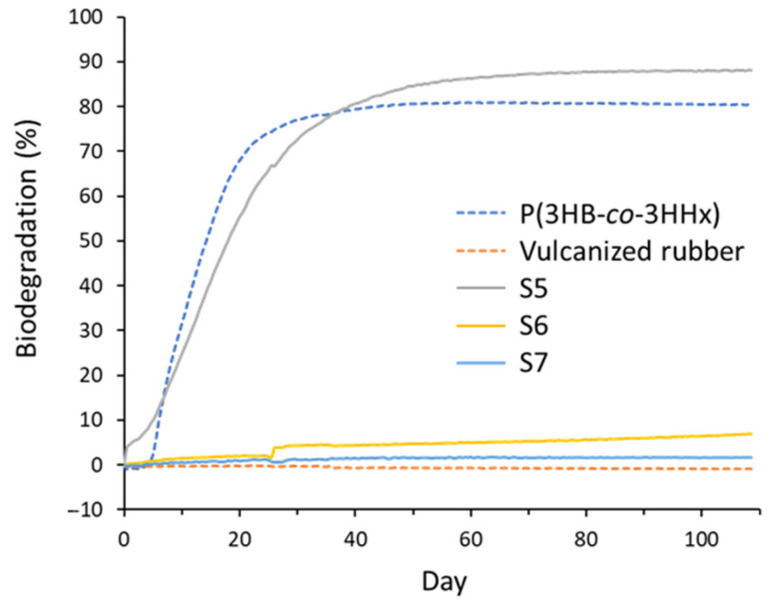
BOD biodegradation curves of P(3HB-*co*-47 mol% 3H5HE) (S5) and vulcanized P(3HB-*co*-47 mol% 3H5HE) (S6 and S7). P(3HB-*co*-5 mol% 3HHx) and vulcanized rubber were also tested as control experiments. The average value of four measurements is shown.

The biodegradation profiles of the samples over 108 days are shown in Figure 6. The samples included non-vulcanized P(3HB-*co*-5 mol% 3HHx), vulcanized rubber (prepared from poly(1-methyl-1-butene-1,4-diyl)), and three P(3HB-*co*-47 mol% 3H5HE) samples (S5, S6, and S7). The theoretical oxygen demand for vulcanized rubber was derived from the empirical formula for pure rubber (C_5_H_8_). In contrast, for S5, S6, and S7, the ThOD values were calculated based on elemental composition and empirical formulas. Over the 108-day test period, S5 (non-vulcanized) and P(3HB-*co*-5 mol% 3HHx) exhibited high biodegradation, reaching 88.1% and 80.4%, respectively. This confirmed the inherent biodegradability of PHA-based materials. In contrast, vulcanized rubber exhibited negligible biodegradation, remaining at approximately 0% throughout the experiment due to the oxygen demand of vulcanized rubber being slightly lower than that without polymer (blank). Among the vulcanized P(3HB-*co*-3H5HE) samples, S6 (containing 5 phr sulfur) exhibited limited biodegradation, reaching 7.0%. Sample S7 (containing 20 phr sulfur) showed even lower biodegradation at only 1.6%. These results indicate that vulcanization significantly hinders the biodegradation process, likely because of the formation of cross-linked networks that are resistant to microbial enzymatic attacks. Nevertheless, both vulcanized PHAs (S6 and S7) demonstrated slightly greater biodegradability than the vulcanized rubber. These findings suggest that, although vulcanization reduces the biodegradability of polymers, vulcanized P(3HB-*co*-3H5HE) retains low biodegradability.

## 4. Discussion

Vulcanization is a widely used method for enhancing the mechanical strength, elasticity, and thermal stability of polymeric materials [24,39]. In this study, the vulcanizable 3HB-based vinyl PHA, P(3HB-*co*-3H5HE), was biosynthesized using sodium 5-hexenoate as the precursor for 3H5HE. By altering the glucose-feeding method during cultivation, the 3H5HE fraction in PHA could be varied from 3 to 47 mol%. Since glucose is a carbon source that causes catabolite repression in cells [40,41], feeding glucose at low concentrations suppresses catabolite repression, making it easier for 3H5HE precursors to be metabolized. A small amount of 3HHx was detected in the PHA copolymers, which was thought to be generated by the biological reduction of the unsaturated bond in 5-hexenoate. All the biosynthesized PHAs were crystalline polymers, exhibiting *T_m_*s in the range of 153–166 °C in the DSC first scan. Crystal formation was also confirmed in P(3HB-*co*-47 mol% 3H5HE), suggesting that these polymers were biosynthesized with a blocky nature. Therefore, the 3HB units were unevenly distributed and crystallized in the polymers. This tendency is similar to that observed in the biosynthesis of other 3HB-based vinyl PHAs as demonstrated in a previous study [18]. Although the culture method used in this study was effective in enhancing the functional properties of PHA, it often resulted in the generation of blocky copolymers.

In this study, the vulcanization reaction was carried out at 155 °C, as MBT is typically used at temperatures ranging from 140 to 160 °C [24]. However, considering the melting point (153–166 °C) of the polymers used in this study, it is likely that some of the crystals did not melt during the vulcanization reaction. The undissolved crystalline domains may act as physical crosslinking points within the vulcanized polymers. The use of crystalline PHAs was different from that in a previous study by Gagnon et al. [24], who demonstrated the vulcanization of amorphous mcl-PHA. On the other hand, the heterogeneous appearance of the films (Figure 2) may be because the crystals did not dissolve during vulcanization.

Vulcanized PHAs did not dissolve in chloroform, indicating that crosslinking occurred between the polymers. DSC analysis revealed that vulcanization led to a slight reduction in the melting temperature of the polymers. This is likely due to the partial disruption of the crystalline regions by the crosslinked networks. The Raman spectroscopy analysis revealed that a fraction of the unsaturated bonds was involved in the cross-link formation. When the amount of sulfur and curing time were changed, a substantial increase in crosslinking was observed. 

For P(3HB-*co*-3 mol% 3H5HE), sulfur vulcanization resulted in decreased mechanical properties such as tensile strength and elongation at break. In contrast, the 19 and 47 mol% 3H5HE copolymers exhibited an increased tensile strength and elongation at break upon sulfur vulcanization. The effect of sulfur vulcanization on the 47 mol% 3H5HE copolymer was investigated in detail. Sample S6 exhibited the most rubber-like properties, showing significantly increased elongation at break (813%), moderate tensile strength (6.3 MPa), and tensile set of 8% after 200% elongation. The toughness increased from 2 to 25 MJ/m^3^ upon sulfur vulcanization. These results suggest that the crosslinking strategy used in this study was effective in producing rubber-like mechanical properties.

On the other hand, crosslinking between polymer chains significantly reduces their susceptibility to microbial degradation. The formation of covalent bonds between polymer chains limits the accessibility of microbial enzymes to ester linkages within the polymer backbone, which are essential for biodegradation. Despite these limitations, the vulcanized PHA examined in this study exhibited a slight degree of biodegradation over time. This can be attributed to the inherently biodegradable nature of the PHA backbone and the possibility of partial enzymatic attack in the less-crosslinked or amorphous regions. In comparison, the vulcanized polyisoprene demonstrated negligible biodegradation. Although crosslinking compromises biodegradability, the bio-based and ester-rich structure of PHA provides a distinct advantage over petroleum-derived elastomers in terms of environmental impact.

## 5. Conclusions

In this study, P(3HB-*co*-3H5HE) was biosynthesized and vulcanized using a sulfur-based system. The introduction of crosslinks via sulfur vulcanization was confirmed using swelling analysis, elemental composition analysis, and Raman spectroscopy. Films of P(3HB-*co*-47 mol% 3H5HE) vulcanized with a moderate sulfur content (5 phr) exhibited the most enhanced elasticity and flexibility, whereas excessive sulfur content (20 phr) led to a decrease in elasticity and flexibility. Biodegradability testing revealed that, although vulcanized P(3HB-*co*-3H5HE) samples exhibited reduced biodegradability compared to non-vulcanized P(3HB-*co*-3H5HE) and P(3HB-*co*-3HHx), they still degraded to a greater extent than vulcanized rubber. This indicates that despite the introduction of crosslinks, the material retained its partial biodegradability. Therefore, sulfur vulcanization is a practical method for enhancing the mechanical properties of unsaturated PHAs while maintaining their degree of environmental degradability. Thus, vulcanized P(3HB-*co*-3H5HE) forms a class of promising candidates for use in sustainable, partially degradable elastomeric materials.

## Figures and Tables

**Table 1:** P(3HB-*co*-3H5HE) biosynthesized in this study.

Glucose Feeding		Copolymer Composition (mol%) ^a^			Molecular Weight ^b^		Polymer Name
Glucose (g/L)	Addition Time (h)	3HB	3HHx	3H5HE	*M_n_* (×10^4^)	PDI	
10 [10 × 1]	0	96.8	0.2	3.0	15.4	3.98	P(3HB-*co*-3 mol% 3H5HE)
11.25 [3.75 × 3]	0, 24, 48	79.7	1.0	19.3	3.98	4.53	P(3HB-*co*-19 mol% 3H5HE)
7.5 [2.5 × 3]	0, 24, 48	52.0	0.8	47.2	8.56	2.33	P(3HB-*co*-47 mol% 3H5HE)

As the 3H5HE precursor, 1.5 g/L sodium 5-hexenoate was supplemented into the culture medium. PHA biosynthesis was conducted by using *E. coli* LS5218 harboring pBBR1*phaP*(D4N)*CJ*_Ac_*AB*_Re_NSDG at 30 °C for 72 h. ^a^ Determined by ^1^H NMR. ^b^ Determined by GPC. PDI: polydispersity index.

**Table 2:** Compound Formulations and Cure Time for Sulfur Vulcanization.

Ingredient	Compound Formulations in Each Sample (phr) ^a^						
	S1	S2	S3	S4	S5	S6	S7
P(3HB-*co*-3 mol% 3H5HE)	100	100	0	0	0	0	0
P(3HB-*co*-19 mol% 3H5HE)	0	0	100	100	0	0	0
P(3HB-*co*-47 mol% 3H5HE)	0	0	0	0	100	100	100
Zinc oxide	0	10	0	10	0	10	10
Stearic acid	0	5	0	5	0	5	5
2-Mercaptobenzothiazole (MBT)	0	10	0	10	0	10	10
Sulfur	0	2	0	2	0	5	20
Curing time (min) ^b^	1	2	1	2	1	2	15

^a^ Per hundred resin. ^b^ Curing at 155 °C.

**Table 4:** Thermal properties of the samples.

Sample	DSC 1st Heating		DSC 2nd Heating				DSC Cooling		TGA		
	*T_m_* (°C)	Δ*H_m_* (J/g)	*T_g_* (°C)	*T_cc_* (°C)	*T_m_* (°C)	Δ*H_m_* (J/g)	*T_c_* (°C)	Δ*H_c_* (J/g)	*T_d_*_5_ (°C) ^a^	*T_d_max_* (°C) ^b^	Residual Mass (%)
S1	166	73	2	52	170	70	92	55	-	-	-
S3	156	29	−5	70	156	24	ND	ND	-	-	-
S5	153	4	−15	60	156	9	ND	ND	251	319	1
S6	153	5	−2	58	154	7	ND	ND	251	312	7
S7	156	4	1	49	155	4	ND	ND	251	331	9

^a^ Temperature at 5% weight loss. ^b^ Maximum degradation temperature. ND: not detectable.

**Table 5:** Cross-linking in P(3HB-*co*-47 mol% 3H5HE) under different conditions.

Sample	Vulcanization Conditions			Peak Area at 1644 cm^−1^ (%) ^b^	Swelling Ratio (%) ^c^	Gel Fraction (%) ^c^	Elemental Composition	
	Sulfur		Curing Time (min)				Sulfur (wt%)	Ash (wt%)
	(phr) ^a^	(wt%)						
S5	0	0	1	100	Dissolved	Dissolved	0	0.5
S6	5	4	2	87.8 ± 0.02	655 ± 172	93 ± 3	2.8	4.0
S7	20	14	15	88.4 ± 0.1	338 ± 26	94 ± 1	7.0	4.4

^a^ Per hundred resin. ^b^ Peak area (1620–1660 cm^−1^) corresponding to C=C stretching in the Raman spectrum. ^c^ Measured with chloroform. Repeated measurements were performed three times (*n* = 3).

## Data Availability

Data are contained within the article. The original contributions presented in this study are included in the article. Further inquiries can be directed to the corresponding author.

## References

[1] Pena C., Castillo T., Garcia A., Millan M., Segura D. (2014). Biotechnological strategies to improve production of microbial poly-(3-hydroxybutyrate): A review of recent research work. Microb. Biotechnol..

[2] Sudesh K., Abe H., Doi Y. (2000). Synthesis, structure and properties of polyhydroxyalkanoates: Biological polyesters. Prog. Polym. Sci..

[3] Koller M., Mukherjee A. (2022). A new wave of industrialization of PHA biopolyesters. Bioengineering.

[4] Das R., Saha N.R., Pal A., Chattopadhyay D., Paul A.K. (2018). Comparative evaluation of physico-chemical characteristics of biopolyesters P(3HB) and P(3HB-*co*-3HV) produced by endophytic *Bacillus cereus* RCL 02. Front. Biol..

[5] Alfano S., Doineau E., Perdrier C., Preziosi-Belloy L., Gontard N., Martinelli A., Grousseau E., Angellier-Coussy H. (2024). Influence of the 3-hydroxyvalerate content on the processability, nucleating and blending ability of poly(3-hydroxybutyrate-*co*-3-hydroxyvalerate)-based materials. ACS Omega.

[6] Tang H.J., Neoh S.Z., Sudesh K. (2022). A review on poly(3-hydroxybutyrate-*co*-3-hydroxyhexanoate) [P(3HB-*co*-3HHx)] and genetic modifications that affect its production. Front. Bioeng. Biotechnol..

[7] Doi Y., Kitamura S., Abe H. (1995). Microbial synthesis and characterization of poly(3-hydroxybutyrate-*co*-3-hydroxyhexanoate). Macromolecules.

[8] Eraslan K., Aversa C., Nofar M., Barletta M., Gisario A., Salehiyan R., Goksu Y.A. (2022). Poly(3-hydroxybutyrate-*co*-3-hydroxyhexanoate) (PHBH): Synthesis, properties, and applications-A review. Eur. Polym. J..

[9] Mizuno S., Nakagawa A., Sakurai T., Miyahara Y., Tsuge T. (2023). Oxidation of methionine-derived 2-hydroxyalkanoate unit in biosynthesized polyhydroxyalkanoate copolymers. Int. J. Biol. Macromol..

[10] Huang P., Okoshi T., Mizuno S., Hiroe A., Tsuge T. (2018). Gas chromatography-mass spectrometry-based monomer composition analysis of medium-chain-length polyhydroxyalkanoates biosynthesized by *Pseudomonas* spp.. Biosci. Biotechnol. Biochem..

[11] Muangwong A., Boontip T., Pachimsawat J., Napathorn S.C. (2016). Medium chain length polyhydroxyalkanoates consisting primarily of unsaturated 3-hydroxy-5-*cis*-dodecanoate synthesized by newly isolated bacteria using crude glycerol. Microb. Cell Fact..

[12] Rodrigues M.F.A., Da Silva L.F., Gomez J.G.C., Valentin H.E., Steinbüchel A. (1995). Biosynthesis of poly(3-hydroxybutyric acid-*co*-3-hydroxy-4-pentenoic acid) from unrelated substrates by *Burkholderia* sp.. Appl. Microbiol. Biotechnol..

[13] Ulmer H.W., Gross R.A., Posada M., Weisbach P., Fuller R.C., Lenz R.W. (1994). Bacterial production of poly(β-hydroxyalkanoates) containing unsaturated repeating units by *Rhodospirillum rubrum*. Macromolecules.

[14] Levine A.C., Heberlig G.W., Nomura C.T. (2016). Use of thiol-ene click chemistry to modify mechanical and thermal properties of polyhydroxyalkanoates (PHAs). Int. J. Biol. Macromol..

[15] Sharma V., Sehgal R., Gupta R. (2021). Polyhydroxyalkanoate (PHA): Properties and modifications. Polymer.

[16] Park W.H., Lenz R.W., Goodwin S. (1998). Epoxidation of bacterial polyesters with unsaturated side chains. I. Production and epoxidation of polyesters from 10-undecenoic acid. Macromolecules.

[17] Anjum A., Zuber M., Zia K.M., Noreen A., Anjum M.N., Tabasum S. (2016). Microbial production of polyhydroxyalkanoates (PHAs) and its copolymers: A review of recent advancements. Int. J. Biol. Macromol..

[18] Miyahara Y., Nakagawa A., Nakata Y., Nomura C.T., Tsuge T. (2025). Polyetylene glycol grafting by thiol–ene reaction for the chemical modification of polyhydroxyalkanoates. Polym. Int..

[19] Imamura T., Kenmoku T., Honma T., Kobayashi S., Yano T. (2001). Direct biosynthesis of poly(3-hydroxyalkanoates) bearing epoxide groups. Int. J. Biol. Macromol..

[20] Bear M.M., Leboucher-Durand M.A., Langlois V., Lenz R.W., Goodwin S., Guérin P. (1997). Bacterial poly-3-hydroxyalkenoates with epoxy groups in the side chains. React. Funct. Polym..

[21] Klongklaew P., Khamjapo P., Sae-Oui P., Jittham P., Loykulnant S., Intiya W. (2023). Characterization and application in natural rubber of *Leucaena* leaf and its extracted products. Polymers.

[22] Yamano M., Yamamoto Y., Saito T., Kawahara S. (2021). Preparation and characterization of vulcanized natural rubber with high stereoregularity. Polymer.

[23] Gagnon K.D., Lenz R.W., Farris R.J., Fuller R.C. (1994). Chemical modification of bacterial elastomers: 1. Peroxide crosslinking. Polymer.

[24] Gagnon K.D., Lenz R.W., Farris R.J., Fuller R.C. (1994). Chemical modification of bacterial elastomers: 2. Sulfur vulcanization. Polymer.

[25] Sukatta U., Rugthaworn P., Seangyen W., Tantaterdtam R., Smitthipong W., Chollakup R. (2021). Prospects for rambutan peel extract as natural antioxidant on the aging properties of vulcanized natural rubber. SPE Polym..

[26] Sato S., Honda Y., Kuwahara M., Watanabe T. (2003). Degradation of vulcanized and nonvulcanized polyisoprene rubbers by lipid peroxidation catalyzed by oxidative enzymes and transition metals. Biomacromolecules.

[27] Cheng Y., Wei Y., Wu H., Zhang T., Li S., Zhu N., Zhang Q., Li W. (2024). Biodegradation of vulcanized natural rubber by enriched bacterial consortia. Chem. Eng. J..

[28] Tong H.S., Kabeb S.M., Abd Hamid H., Zulkifli F.H. (2025). A review of biodegradability of natural rubber products: Physicochemical, thermal and mechanical properties. Int. J. Biol. Macromol..

[29] Suzuki M., Tachibana Y., Kasuya K.I. (2021). Biodegradability of poly(3-hydroxyalkanoate) and poly(ε-caprolactone) via biological carbon cycles in marine environments. Polym. J..

[30] Hachisuka S.I., Sakurai T., Mizuno S., Kosuge K., Endo S., Ishii-Hyakutake M., Miyahara Y., Yamazaki M., Tsuge T. (2023). Isolation and characterization of polyhydroxyalkanoate-degrading bacteria in seawater at two different depths from Suruga Bay. Appl. Environ. Microbiol..

[31] Narancic T., Verstichel S., Reddy Chaganti S., Morales-Gamez L., Kenny S.T., De Wilde B., Padamati R.B., O’Connor K.E. (2018). Biodegradable plastic blends create new possibilities for end-of-life management of plastics but they are not a panacea for plastic pollution. Environ. Sci. Technol..

[32] Watanabe Y., Ishizuka K., Furutate S., Abe H., Tsuge T. (2015). Biosynthesis and characterization of novel poly(3-hydroxybutyrate-*co*-3-hydroxy-2-methylbutyrate): Thermal behavior associated with α-carbon methylation. RSC Adv..

[33] Ushimaru K., Watanabe Y., Hiroe A., Tsuge T. (2015). A single-nucleotide substitution in phasin gene leads to enhanced accumulation of polyhydroxyalkanoate (PHA) in *Escherichia coli* harboring *Aeromonas caviae* PHA biosynthetic operon. J. Gen. Appl. Microbiol..

[34] Spratt S.K., Ginsburgh C.L., Nunn W.D. (1981). Isolation and genetic characterization of *Escherichia coli* mutants defective in propionate metabolism. J. Bacteriol..

[35] Wang H., Liu J., Fan X., Ren J., Liu Q., Kong B. (2022). Fabrication, characterisation, and application of green crosslinked sodium alginate hydrogel films by natural crab-shell powders to achieve drug sustained release. LWT.

[36] Mierzati M., Sakurai T., Ishii-Hyakutake M., Miyahara Y., Nomura C.T., Taguchi S., Abe H., Tsuge T. (2023). Biosynthesis, characterization, and biodegradation of elastomeric polyhydroxyalkanoates consisting of α-dimethylated monomer units. Mater. Today Sustain..

[37] Furukawa T., Sato H., Murakami R., Zhang J., Noda I., Ochiai S., Ozaki Y. (2006). Raman microspectroscopy study of structure, dispersibility, and crystallinity of poly(hydroxybutyrate)/poly(l-lactic acid) blends. Polymer.

[38] Sato H., Dybal J., Murakami R., Noda I., Ozaki Y. (2005). Infrared and Raman spectroscopy and quantum chemistry calculation studies of C–H⋯O hydrogen bondings and thermal behavior of biodegradable polyhydroxyalkanoate. J. Mol. Struct..

[39] Bokobza L. (2018). Natural rubber nanocomposites: A review. Nanomaterials.

[40] Stülke J., Hillen W. (1999). Carbon catabolite repression in bacteria. Curr. Opin. Microbiol..

[41] Pavoncello V., Barras F., Bouveret E. (2022). Degradation of exogenous fatty acids in *Escherichia coli*. Biomolecules.

